# Randomization Does Not Help Much, Comparability Does

**DOI:** 10.1371/journal.pone.0132102

**Published:** 2015-07-20

**Authors:** Uwe Saint-Mont

**Affiliations:** Nordhausen University of Applied Sciences, Nordhausen, Germany; National Taiwan University, TAIWAN

## Abstract

According to R.A. Fisher, randomization “relieves the experimenter from the anxiety of considering innumerable causes by which the data may be disturbed.” Since, in particular, it is said to control for known and unknown nuisance factors that may considerably challenge the validity of a result, it has become very popular. This contribution challenges the received view. First, looking for quantitative support, we study a number of straightforward, mathematically simple models. They all demonstrate that the optimism surrounding randomization is questionable: In small to medium-sized samples, random allocation of units to treatments typically *yields* a considerable imbalance between the groups, i.e., confounding due to randomization is the rule rather than the exception. In the second part of this contribution, the reasoning is extended to a number of traditional arguments in favour of randomization. This discussion is rather non-technical, and sometimes touches on the rather fundamental Frequentist/Bayesian debate. However, the result of this analysis turns out to be quite similar: While the contribution of randomization remains doubtful, comparability contributes much to a compelling conclusion. Summing up, classical experimentation based on sound background theory and the systematic construction of exchangeable groups seems to be advisable.

## 1 The logic of the experiment

Randomization, the allocation of subjects to experimental conditions via a random procedure, was introduced by eminent statistician R.A. Fisher [1]. Arguably, it has since become the most important statistical technique. In particular, statistical experiments are defined by the use of randomization [2, 3], and many applied fields, such as *evidence based medicine*, draw a basic distinction between randomized and non-randomized evidence.

In order to explain randomization’s eminent role, one may refer to the logic of the experiment, largely based on J. S. Mill’s *method of difference*[4]: If one compares two groups of subjects (Treatment *T* versus Control *C*, say) and observes a salient contrast in the end (e.g. ${\bar{X}}_{T}>{\bar{X}}_{C}$), that difference must be due to the experimental manipulation—IF the groups were equivalent at the very beginning of the experiment.

In other words, since the difference between treatment and control (i.e. the experimental manipulation) is the only perceivable reason that can explain the variation in the observations, it must be the cause of the observed effect (the difference in the end). The situation is quite altered, however, if the two groups already differed substantially at the beginning. Then (see Table 1 below), there are two possible explanations of an effect:

**Table 1:** Mill’s logic.

Start of Experiment	*T*	=	*C*	*T*	≠	*C*
Intervention	Yes		No	Yes		No
End of Experiment (Observed Effect)	${\bar{X}}_{T}$	>	${\bar{X}}_{C}$	${\bar{X}}_{T}$	>	${\bar{X}}_{C}$
Conclusion	Intervention caused the effect			Intervention OR Prior Difference between the groups caused the effect		

## 2 Comparability

Thus, for the logic of the experiment, it is of paramount importance to ensure equivalence of the groups at the beginning of the experiment. The groups, or even the individuals involved, must not be systematically different; one has to compare like with like. Alas, in the social sciences exact equality of units, e.g. human individuals, cannot be maintained. Therefore one must settle for *comparable* subjects or groups (*T* ≈ *C*).

### 2.1 Defining comparability

In practice, it is straightforward to define comparability with respect to the features or properties of the experimental units involved. In a typical experimental setup, statistical units (e.g. persons) are represented by their corresponding vectors of attributes (properties, variables) such as gender, body height, age, etc.

If the units are almost equal in as many properties as possible, they should be comparable, i.e., the remaining differences shouldn’t alter the experimental outcome substantially. However, since, in general, vectors have to be compared, there is not a single measure of similarity. Rather, there are quite a lot of measures available, depending on the kind of data at hand. An easily accessible and rather comprehensive overview may be found here: reference.wolfram.com/mathematica/guide/DistanceAndSimilarityMeasures.html

As an example, suppose a unit *i* is represented by a binary vector **a**_*i*_ = (*a*_*i*1_, …, *a*_*im*_). The Hamming distance *d*(⋅,⋅) between two such vectors is the number of positions at which the corresponding symbols are different. In other words, it is the minimum number of substitutions required to change one vector into the other. Let **a**_1_ = (0,0,1,0), **a**_2_ = (1,1,1,0), and **a**_3_ = (1,1,1,1). Therefore *d*(**a**_1_,**a**_2_) = 2, *d*(**a**_1_,**a**_3_) = 3, *d*(**a**_2_,**a**_3_) = 1, and *d*(**a**_*i*_,**a**_*i*_) = 0. Having thus calculated a reasonable number for the “closeness” of two experimental units, one next has to consider what level of deviance from perfect equality may be tolerable.

Due to this, coping with similarities is a tricky business. Typically many properties (covariates) are involved and conscious (subjective) judgement seems to be inevitable. An even more serious question concerns the fact that relevant factors may not have been recorded or might be totally unknown. In the worst case, similarity with respect to some known factors has been checked, but an unnoticed nuisance variable is responsible for the difference between the outcome in the two groups.

Moreover, comparability depends on the phenomenon studied. A clearly visible difference, such as gender, is likely to be important with respect to life expectancy, and can influence some physiological and psychological variables such as height or social behaviour, but it is independent of skin color or blood type. In other words, experimental units do not need to be twins in any respect; it suffices that they be similar with respect to the outcome variable under study.

Given a unique sample it is easy to think about a *reference set* of other samples that are alike in all relevant respects to the one observed. However, even Fisher could not give these words a precise formal meaning [5]. Thus De Finetti [6] proposed *exchangeability*, i.e. “instead of judging whether two groups are similar, the investigator is instructed to imagine a hypothetical *exchange* of the two groups … and then judge whether the observed data under the swap would be distinguishable from the actual data” (see [7], p. 196). Barnard [8] gives some history on this idea and suggests the term *permutability*, “which conveys the idea of replacing one thing by another similar thing.” Nowadays, epidemiologists say that “the effect of treatment is *unconfounded* if the treated and untreated groups resemble each other in all relevant features” [7], p. 196.

### 2.2 Experimental techniques to achieve comparability

There are a number of strategies to achieve comparability. Starting with the experimental units, it is straightforward to *match* similar individuals, i.e., to construct pairs of individuals that are alike in many (most) respects. Looking at the group level (*T* and *C*), another straightforward strategy is to *balance* all relevant variables when assigning units to groups. Many approaches of this kind are discussed in [9], minimization being the most prominent among them. Treasure and MacRae [10] explain:

In our study of aspirin versus placebo … we chose age, sex, operating surgeon, number of coronary arteries affected, and left ventricular function. But in trials in other diseases those chosen might be tumour type, disease stage, joint mobility, pain score, or social class.

At the point when it is decided that a patient is definitely to enter a trial, these factors are listed. The treatment allocation is then made, not purely by chance, but by determining in which group inclusion of the patient would minimise any differences in these factors. Thus, if group A has a higher average age and a disproportionate number of smokers, other things being equal, the next elderly smoker is likely to be allocated to group B. The allocation may rely on minimisation alone, or still involve chance but ‘with the dice loaded’ in favour of the allocation which minimises the differences.

However, apart from being cumbersome and relying on the experimenter’s expertise (in particular in choosing and weighing the factors), these strategies are always open to the criticism that unknown nuisance variables may have had a substantial impact on the result. Therefore Fisher [1], pp. 18–20, advised strongly against treating every conceivable factor explicitly. Instead, he taught that “the random choice of the objects to be treated in different ways [guarantees] the validity of the test of significance … against corruption by the causes of disturbance which have not been eliminated.” More explicitly, Berger [11], pp. 9–10, explains:

The idea of randomization is to overlay a sequence of units (subjects, or patients) onto a sequence of treatment conditions. If neither sequence can influence the other, then there should be no bias in the assignment of the treatments, and the comparison groups should be comparable.

### 2.3 Randomization vs. comparability

Historically, Fisher’s idea proved to be a great success [12]. Randomized controlled trials (RCTs), as much as the randomized evidence they produce became the gold standard in a number of fields, and watchwords highlighting randomization’s leading part spread, e.g. “randomization controls for all possible confounders, known and unknown.”

Nevertheless, there have always been reservations about randomization. Putting the basic logic of the experiment in first place, randomization is a lower-ranking tool, employed towards the end of comparability. Moreover, it would be rather problematic if randomization failed to reliably yield similar groups, since non-comparable groups offer a straightforward alternative explanation, undermining experimental validity. To this end, Greenland [13], see Table 2, came up with “the smallest possible controlled trial” illustrating that randomization does not prevent confounding:

**Table 2:** Greenland’s example.

*P*_1_		*P*_2_	Start of Experiment	*P*_2_		*P*_1_
Yes		No	Intervention	Yes		No
${\bar{X}}_{T}$	>	${\bar{X}}_{C}$	End of Experiment	${\bar{X}}_{T}$	>	${\bar{X}}_{C}$

That is, he flips a coin once in order to assign two patients to *T* and *C*, respectively: If heads, the first patient is assigned to *T*, and the second to *C*; if tails, the first patient is assigned to *C*, and the second to *T*. Suppose ${\bar{X}}_{T}>{\bar{X}}_{C}$, what is the reason for the observed effect? Due to the experimental design, there are two alternatives: either the treatment condition differed from the control condition, or patient *P*_1_ was not comparable to patient *P*_2_. However, as each patient is only observed under either the treatment or control (the left hand side or the right hand side of the above table), one cannot distinguish between the patient’s and the treatment’s impact on the observed result. Therefore Greenland concludes that “no matter what the outcome of randomization, the study will be completely confounded.” This effect has been observed on many occasions, for similar remarks see [10, 14–22]. In total generality, Berger [11], p. 9, states:

While it is certainly true that randomization is used for the purpose of ensuring comparability between or among comparison groups, … it is categorically not true that this goal is achieved.

Suppose the patients are perfect twins with the exception of a single difference. Then Greenland’s example shows that randomization cannot even balance a *single* nuisance factor. To remedy the defect, it is straightforward to increase *n*. However, no quantitative advice is given here or elsewhere. Thus it should be worthwhile studying a number of explicit and straightforward models, *quantifying* the effects of randomization. Moreover, quite early, statisticians—in particular of the Bayesian persuasion—put forward several rather diverse arguments against randomization [23–29]. At this point, it is not necessary to delve into delicate philosophical matters or the rather violent Bayesian-Frequentist debate (however, see Section 5), since fairly elementary probabilistic arguments suffice to demonstrate that the above criticism hits its target: By its very nature a random mechanism provokes fluctuations in the composition of *T* and *C*, making these groups (rather often) non-comparable.

The subsequent reasoning has the advantage of being straightforward, mathematical, and not primarily “foundational”. Its flavour is Bayesian in the sense that we are comparing the *actual groups* produced by randomization which is the “posterior view” preferred by that school. At the same time its flavour is Frequentist, since we are focusing on the properties of a certain random *procedure* which is the “design view” preferred by this school. There are not just two, but (at least) three, competing statistical philosophies, and “in many ways the Bayesian and frequentist philosophies stand at opposite poles from each other, with Fisher’s ideas being somewhat of a compromise” [30]. Since randomization is a Fisherian proposal, a neutral quantitative analysis of his approach seems to be appropriate, acceptable to all schools, and, in a sense, long overdue. To a certain degree, philosophy is a matter of opinion. However, it is difficult to argue with mathematical facts of actual performance (see [31], p. xxii).

## 3 Random confounding

The overall result of the following calculations is thus [32]:

Despite randomization, imbalance in prognostic factors as a result of chance (chance imbalance) may still arise, and with small to moderate sample sizes such imbalance may be substantial.

### 3.1 Dichotomous factors

Suppose there is a nuisance factor *X* taking the value 1 if present and 0 if absent. One may think of *X* as a genetic aberration, a medical condition, a psychological disposition or a social habit. Assume that the factor occurs with probability *p* in a certain person (independent of anything else). Given this, 2*n* persons are randomized into two groups of equal size by a chance mechanism independent of *X*.

Let *S*_1_ and *S*_2_ count the number of persons with the trait in the first and the second group respectively. *S*_1_ and *S*_2_ are independent random variables, each having a binomial distribution with parameters *n* and *p*. A natural way to measure the extent of imbalance between the groups is *D* = *S*_1_ − *S*_2_. Obviously, *ED* = 0 and

$$\sigma^{2}\left(D\right)=\sigma^{2}\left(S_{1}\right)+\sigma^{2}\left(-S_{2}\right)=2\sigma^{2}\left(S_{1}\right)=2np\left(1-p\right).$$

Iff *D* = 0, the two groups are perfectly balanced with respect to factor *X*. In the worst case ∣*D*∣ = *n*, that is, in one group all units possess the characteristic, whereas it is completely absent in the other. For fixed *n*, let the two groups be comparable if ∣*D*∣ ≤ *n*/*i* with some *i* ∈ {1, …, *n*}. Iff *i* = 1, the groups will always be considered comparable. However, the larger *i*, the smaller the number of cases we classify as comparable. In general, *n*/*i* defines a proportion of the range of ∣*D*∣ that seems to be acceptable. Since *n*/*i* is a positive number, and *S*_1_ = *S*_2_ ⇔ ∣*D*∣ = 0, the set of comparable groups is never empty.

Given some constant *i*(< *n*), the value *n*/*i* grows at a linear rate in *n*, whereas $\sigma (D)=\sqrt{2np(1-p)}$ grows much more slowly. Due to continuity, there is a single point *n*(*i*, *k*), where the line intersects with *k* times the standard deviation of *D*. Beyond this point, i.e. for all *n* ≥ *n*(*i*, *k*), at least as many realizations of ∣*D*∣ will be within the acceptable range [0, *n*/*i*]. Straightforward algebra gives,

$$n_{p}\left(i,k\right)=2p\left(1-p\right)i^{2}k^{2}.$$

#### Examples

**Fig 1:**
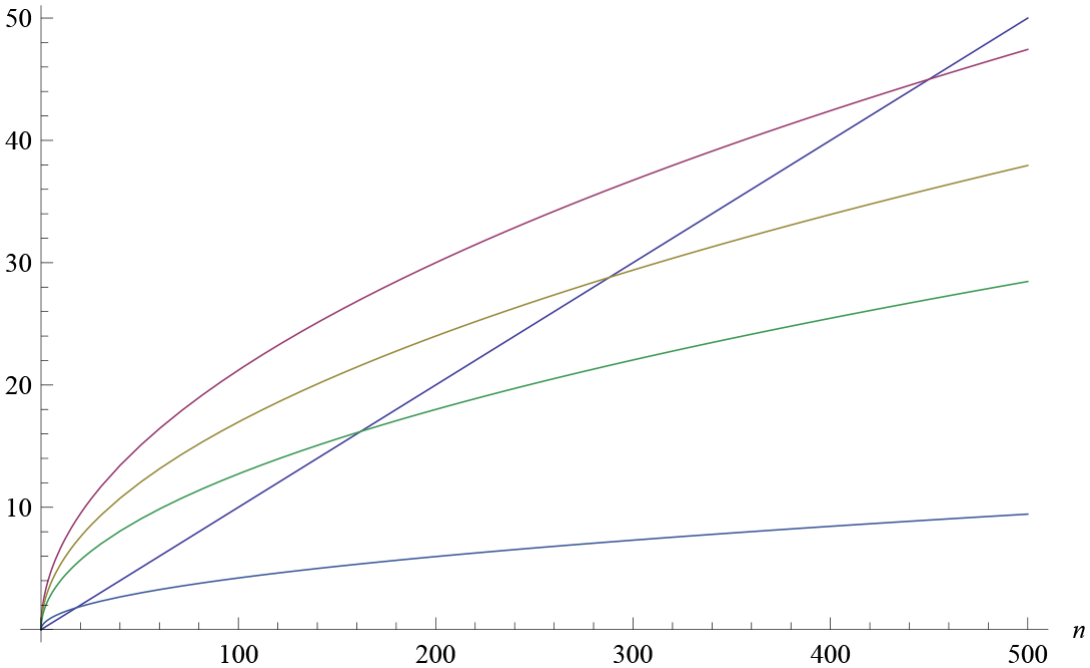
The linear function *n*/10, and from above to below *f*_*p*_(*n*) for $p=\frac{1}{2},\frac{1}{5},\frac{1}{10},$ and $\frac{1}{100}$.

A typical choice could be *i* = 10 and *k* = 3, which specifies the requirement that most samples be located within a rather tight acceptable range. In this case, one has to consider the functions *n*/10 and $f_{p}(n)=3\sqrt{2p(1-p)n}$. These functions of *n* are shown in the following figure (Fig 1):

Thus, depending on *p*, the following numbers of subjects are needed per group (and twice this number altogether):

$$\begin{array}{ccccc} p & i & k & n_{p}\left(i,k\right) & 2\cdot n_{p}\left(i,k\right)\\ 1/2 & 10 & 3 & 450 & 900\\ 1/5 & 10 & 3 & 288 & 576\\ 1/10 & 10 & 3 & 162 & 324\\ 1/100 & 10 & 3 & 18 & 36\; \end{array}$$

Relaxing the criterion of comparability (i.e. a smaller value of *i*) decreases the number of subjects necessary:

$$\begin{array}{cccc} p & i & k & n_{p}\left(i,k\right)\\ 1/2 & 5 & 3 & 113\\ 1/5 & 5 & 3 & 72\\ 1/10 & 5 & 3 & 41\\ 1/100 & 5 & 3 & 5\; \end{array}$$

The same happens if one decreases the number of standard deviations *k*:

$$\begin{array}{cccc} p & i & k & n_{p}\left(i,k\right)\\ 1/2 & 10 & 2 & 200\\ 1/5 & 10 & 2 & 128\\ 1/10 & 10 & 2 & 72\\ 1/100 & 10 & 2 & 8\; \end{array}$$

This shows that randomization works, if the number of subjects ranges in the hundreds or if the probability *p* is rather low. (By symmetry, the same conclusion holds if *p* is close to one.) Otherwise there is hardly any guarantee that the two groups will be comparable. Rather, they will differ considerably due to random fluctuations.

The distribution of *D* is well known ([33], pp. 142–143). For *d* = −*n*, …, *n*,

P(D=d)=∑max(0,d)≤y≤min(n,n+d)(ny)(ny-d)p2y-d(1-p)2n-2y+d

Therefore, it is also possible to compute the probability *q* = *q*(*i*, *n*, *p*) that two groups, constructed by randomization, will be comparable. If *i* = 5, i.e., if one fifth of the range of ∣*D*∣ is judged to be comparable, we obtain:

$$\begin{array}{ccc} p & n & q(i,n,p)\\ 1/2 & 5 & 0.66\\ 1/2 & 10 & 0.74\\ 1/2 & 25 & 0.88\\ 1/2 & 50 & 0.96\; \end{array}\;\begin{array}{ccc} p & n & q(i,n,p)\\ 1/10 & 5 & 0.898\\ 1/10 & 10 & 0.94\\ 1/10 & 25 & 0.98999\\ 1/10 & 50 & 0.999\; \end{array}\;\begin{array}{ccc} p & n & q(i,n,p)\\ 1/100 & 5 & 0.998\\ 1/100 & 10 & 0.9997\\ 1/100 & 25 & 0.999999\\ 1/100 & 50 & 1\; \end{array}$$

Thus, it is rather difficult to control a factor that has a probability of about 1/2 in the population. However, even if the probability of occurrence is only about 1/10, one needs more than 25 people per group to have reasonable confidence that the factor has not produced a substantial imbalance.

#### Several factors

The situation becomes worse if one takes more than one nuisance factor into account. Given *m* independent binary factors, each of them occurring with probability *p*, the probability that the groups will be balanced with respect to all nuisance variables is *q* ^*m*^. Numerically, the above results yield:

$$\begin{array}{cccccc} p & n & q & q^{2} & q^{5} & q^{10}\\ 1/2 & 5 & 0.66 & 0.43 & 0.12 & 0.015\\ 1/2 & 10 & 0.74 & 0.54 & 0.217 & 0.047\\ 1/2 & 25 & 0.88 & 0.78 & 0.53 & 0.28\\ 1/2 & 50 & 0.96 & 0.93 & 0.84 & 0.699 \end{array}$$

$$\begin{array}{cccccc} p & n & q & q^{2} & q^{5} & q^{10}\\ 1/10 & 5 & 0.898 & 0.807 & 0.58 & 0.34\\ 1/10 & 10 & 0.94 & 0.88 & 0.74 & 0.54\\ 1/10 & 25 & 0.98999 & 0.98 & 0.95 & 0.90\\ 1/10 & 50 & 0.999 & 0.9989 & 0.997 & 0.995 \end{array}$$

$$\begin{array}{cccccc} p & n & q & q^{2} & q^{5} & q^{10}\\ 1/100 & 5 & 0.998 & 0.996 & 0.99 & 0.98\\ 1/100 & 10 & 0.9998 & 0.9996 & 0.999 & 0.9979\\ 1/100 & 25 & 0.9999998 & 0.9999995 & 0.999999 & 0.999998\\ 1/100 & 50 & 1 & 1 & 1 & 1 \end{array}$$

Accordingly, given *m* independent binary factors, each occurring with probability *p*_*j*_ (and corresponding *q*_*j*_ = *q*(*i*, *n*, *p*_*j*_)), the probabilities closest to 1/2 will dominate 1 − *q*_1_⋯*q*_*m*_, which is the probability that the two groups are not comparable due to an imbalance in at least one variable. In a typical study with 2*n* = 100 persons, for example, it does not matter if there are one, two, five or even ten factors, if each of them occurs with probability of 1/100. However, if some of the factors are rather common (e.g. 1/5 < *p*_*j*_ < 4/5), this changes considerably. In a smaller study with fewer than 2*n* = 50 participants, a few such factors suffice to increase the probability that the groups constructed by randomization won’t be comparable to 50%. With only a few units per group, one can be reasonably sure that some undetected, but rather common, nuisance factor(s) will make the groups non-comparable. Altogether our conclusion based on an explicit quantitative analysis coincides with the qualitative argument given by [23], p. 91 (my emphasis):

Suppose we had, say, thirty fur-bearing animals of which some were junior and some senior, some black and some brown, some fat and some thin, some of one variety and some of another, some born wild and some in captivity, some sluggish and some energetic, and some long-haired and some short-haired. It might be hard to base a convincing assay of a pelt-conditioning vitamin on an experiment with these animals, for every subset of fifteen might well contain nearly all of the animals from one side or another of one of the important dichotomies …

Thus contrary to what I think I was taught, and certainly used to believe, *it does not seem possible to base a meaningful experiment on a small heterogenous group*.

#### Interactions

The situation deteriorates considerably if there are interactions between the variables that may also yield convincing alternative explanations for an observed effect. It is possible that all factors considered in isolation are reasonably balanced (which is often checked in practice), but that a certain combination of them affects the observed treatment effect. For the purpose of illustration (see Table 3 below), suppose four persons (being young or old, and male or female) are investigated:

**Table 3:** Example of an interaction.

T	C
Old Man	Old Woman
Young Woman	Young Man

Although gender and (dichotomized) age are perfectly balanced between *T* and *C*, the young woman has been allocated to the first group. Therefore a property of young women (e.g. pregnancy) may serve as an explanation for an observed effect, e.g. ${\bar{X}}_{T}>{\bar{X}}_{C}$.

Given *m* factors, there are *m*(*m* − 1)/2 possible interactions between just two of the factors, and (mν) possible interactions between *ν* of them. Thus, there is a high probability that some considerable imbalance occurs in at least one of these numerous interactions, in small groups in particular. For a striking early numerical study see [34]. Detected or undetected, such imbalances provide excellent alternative explanations of an observed effect.

In the light of this, one can only hope for some ‘benign’ dependence structure among the factors, i.e., a reasonable balance in one factor improving the balance in (some of) the others. Given such a tendency, a larger number of nuisance factors may be controlled, since it suffices to focus on only a few. Independent variables possess a ‘neutral’ dependence structure in that the balance in one factor does not influence the balance in others. Yet, there may be a ‘malign’ dependence structure, such that balancing one factor tends to actuate imbalances in others. We will make this argument more precise in Section 4. However, a concrete example will illustrate the idea: Given a benign dependence structure, catching one cow (balancing one factor) makes it easier to catch others. Therefore it is easy to lead a herd into an enclosure: Grab some of the animals by their horns (balance some of the factors) and the others will follow. However, in the case of a malign dependence structure the same procedure tends to stir up the animals, i.e., the more cows are caught (the more factors are being balanced), the less controllable the remaining herd becomes.

### 3.2 Ordered random variables

In order to show that our conclusions do not depend on some specific model, let us next consider ordered random variables. To begin with, look at four units with ranks 1 to 4. If they are split into two groups of equal size, such that the best (1) and the worst (4) are in one group, and (2) and (3) are in the other, both groups have the same rank sum and are thus comparable. However, if the best and the second best constitute one group and the third and the fourth the other group, their rank sums (3 versus 7) differ by the maximum amount possible, and they do not seem to be comparable. If the units with ranks 1 and 3 are in the first group and the units with ranks 2 and 4 are in the second one, the difference in rank sums is ∣6 − 4∣ = 2 and it seems to be a matter of personal judgement whether or not one thinks of them as comparable.

Given two groups, each having *n* members, the total sum of ranks is *r* = 2*n*(2*n*+1)/2 = *n*(2*n*+1). If, in total analogy to the last section, *S*_1_ and *S*_2_ are the sum of the ranks in the first and the second group, respectively, *S*_2_ = *r* − *S*_1_. Therefore it suffices to consider *S*_1_, which is the test statistic of Wilcoxon’s test. Again, a natural way to measure the extent of imbalance between the groups is *D* = *S*_1_ − *S*_2_ = 2*S*_1_ − *r*. Like before *ED* = 0, and because *σ* ^2^(*S*_1_) = *n* ^2^(2*n*+1)/12 we have

$$\sigma^{2}\left(D\right)=4\sigma^{2}\left(S_{1}\right)=n^{2}\left(2n+1\right)/3$$

Moreover, *n*(*n*+1)/2 ≤ *S*_*j*_ ≤ *n*(3*n*+1)/2 (*j* = 1,2) yields −*n* ^2^ ≤ *D* ≤ *n* ^2^. Thus, in this case, *n* ^2^/*i* (*i* ∈ {1, …, *n* ^2^}) determines a proportion of the range of ∣*D*∣ that may be used to define comparability. Given a fixed *i*(< *n* ^2^), the quantity *n* ^2^/*i* is growing at a quadratic rate in *n*, whereas $\sigma (D)=n\sqrt{(2n+1)/3}$ is growing at a slower pace. Like before, there is a single point *n*(*i*, *k*), where *n* ^2^/*i* = *kσ*(*D*). Straightforward algebra gives,

$$n\left(i,k\right)=ik\left(ik+\sqrt{{\left(ik\right)}^{2}+3}\right)/3.$$

Again, we see that large numbers of observations are needed to ensure comparability:

$$\begin{array}{ccc} i & k & n(i,k)\\ 10 & 3 & 501\\ 5 & 3 & 156\\ 10 & 2 & 267 \end{array}$$

As before, it is possible to work with the distribution of *D* explicitly. That is, given *i* and *n*, one may calculate the probability *q* = *q*(*i*, *n*) that two groups, constructed by randomization, are comparable. If ∣*D*∣ ≤ *n* ^2^/*i* is considered comparable, it is possible to obtain, using the function pwilcox() in R:

ni510255010030.580.780.960.996150.450.560.780.920.99100.160.320.450.610.78

These results for ordered random variables are perfectly in line with the conclusions drawn from the binary model. Moreover, the same argument as before shows that the situation becomes (considerably) worse if several factors may influence the final result.

### 3.3 A continuous model

Finally, we consider a continuous model. Suppose there is just one factor *X* ∼ *N*(*μ*, *σ*). One may think of *X* as a normally distributed personal ability, person *i* having individual ability *x*_*i*_. As before, assume that 2*n* persons are randomized into two groups of equal size by a chance mechanism independent of the persons’ abilities.

Suppose that also in this model *S*_1_ and *S*_2_ measure the total amount of ability in the first and the second group respectively. Obviously, *S*_1_ and *S*_2_ are independent random variables, each having a normal distribution $N(n\mu ,\sqrt{n}\sigma )$. A straightforward way to measure the *absolute* extent of imbalance between the groups is

$$D=S_{1}-S_{2}=\sum_{i=1}^{n}X_{1,i}-\sum_{i=1}^{n}X_{2,i}=\sum_{i=1}^{n}\left(X_{1,i}-X_{2,i}\right).$$ (1)

Due to independence, obviously $D∼N(0,\sqrt{2n}\sigma )$.

Let the two groups be comparable if ∣*D*∣ ≤ *lσ*, i.e., if the difference between the abilities assembled in the two groups does not differ by more than *l* standard deviations of the ability *X* in a single unit. The larger *l*, the more cases are classified as comparable. For every fixed *l*, *lσ* is a constant, whereas $\sigma (D)=\sqrt{2n}\sigma$ is growing slowly. Owing to continuity, there is yet another single point *n*(*l*), where $l\sigma =\sigma (D)=\sqrt{2n}\sigma$. Straightforward algebra gives,

$$l\leq \sqrt{2n}\Leftrightarrow 2n\geq l^{2}.$$

In particular, we have:

$$\begin{array}{cccccc} l & 1 & 2 & 3 & 5 & 10\\ n & 1 & 2 & 5 & 13 & 50 \end{array}$$

In other words, the two groups become non-comparable very quickly. It is almost impossible that two groups of 500 persons each, for example, could be close to one another with respect to total (absolute) ability.

However, one may doubt if this measure of non-comparability really makes sense. Given two teams with a hundred or more subjects, it does not seem to matter whether the total ability in the first one is within a few standard deviations of the other. Therefore it is reasonable to look at the *relative* advantage of group 1 with respect to group 2, i.e. *Q* = *D*/*n*. Why divide by *n* and not by some other function of *n*? First, due to Eq (1), exactly *n* comparisons *X*_1, *i*_ − *X*_2, *i*_ have to be made. Second, since

$$Q=\sum_{i=1}^{n}X_{1,i}/n-\sum_{i=1}^{n}X_{2,i}/n={\bar{X}}_{T}-{\bar{X}}_{C},$$

*Q* may be interpreted in a natural way, i.e., being the difference between the typical (mean) representative of group 1 (treatment) and the typical representative of group 2 (control). A straightforward calculation yields $Q∼N(0,\sigma \sqrt{2/n})$.

Let the two groups be comparable if ∣*Q*∣ ≤ *lσ*. If one wants to be reasonably sure (three standard deviations of *Q*) that comparability holds, we have $l\sigma \geq 3\sigma \sqrt{2/n}\Leftrightarrow n\geq 18/l^{2}$. Thus, at least the following numbers of subjects are required per group:

$$\begin{array}{ccccccc} l & 5 & 2 & 1 & 1/2 & 1/4 & 1/8\\ n & 1 & 5 & 18 & 72 & 288 & 1152 \end{array}$$

If one standard deviation is considered a large effect [35], three dozen subjects are needed to ensure that such an effect will not be produced by chance. To avoid a small difference between the groups due to randomization (one quarter of a standard deviation, say), the number of subjects needed goes into the hundreds.

In general, if *k* standard deviations of *Q* are desired, we have,

$$n\geq 2k^{2}/l^{2}.$$

Thus, for *k* = 1,2 and 5, the following numbers of subjects *n*_*k*_ are required in each group:

$$\begin{array}{ccccccc} l & 5 & 2 & 1 & 1/2 & 1/4 & 1/8\\ n_{1} & 1 & 1 & 2 & 8 & 32 & 128\\ n_{2} & 1 & 2 & 8 & 32 & 128 & 512\\ n_{5} & 2 & 13 & 50 & 200 & 800 & 3200 \end{array}$$

These are just the results for one factor. As before, the situation deteriorates considerably if one sets out to control several nuisance variables by means of randomization.

### 3.4 Intermediate conclusions

The above models have deliberately been kept as simple as possible. Their results are straightforward and they agree: If *n* is small, it is almost impossible to control for a trait that occurs frequently at the individual level, or for a larger number of confounders, via randomization. It is of paramount importance to understand that random fluctuations lead to considerable differences between small or medium-sized groups, making them very often non-comparable, thus undermining the basic logic of experimentation. That is, ‘blind’ randomization does not create equivalent groups, but rather *provokes* imbalances and subsequent artifacts. Even in larger samples one needs considerable luck to succeed in creating equivalent groups: *p* close to 0 or 1, a small number of nuisance factors *m*, or a favourable dependence structure that balances all factors, including their relevant interactions, if only some crucial factors are to be balanced by chance.

## 4 Unknown factors

Had the trial not used random assignment, had it instead assigned patients one at a time to balance [some] covariates, then the balance might well have been better [for those covariates], but there would be no basis for expecting other unmeasured variables to be similarly balanced ([2], p. 21)

This is a straightforward and popular argument in favour of randomization. Since randomization treats known and unknown factors alike, it is quite an asset that one may thus infer from the observed to the unobserved without further assumptions. However, this argument backfires immediately since, for exactly the same reason, an imbalance in an observed variable cannot be judged as harmless. Quite the contrary: With random assignment there is some basis for expecting other unmeasured variables to be similarly unbalanced. An observed imbalance *hints at* further undetectable imbalances in unobserved variables.

Moreover, treating known and unknown factors equivalently is cold comfort compared to the considerable amount of imbalance evoked by randomization. Fisher’s favourite method always comes with the cost that it introduces additional variability, whereas a systematic schema at least balances known factors. In subject areas haunted by heterogeneity it seems intuitively right to deliberately work in favour of comparability, and rather odd to introduce further variability.

In order to sharpen these qualitative arguments, let us look at an observed factor *X*, an unobserved factor *Y*, and their dependence structure in more detail. Without loss of generality let all functions *d* be positive in the following. Having constructed two groups of equal size via randomization, suppose $d_{R}(X)={\bar{X}}_{T}-{\bar{X}}_{C}>0$ is the observed difference between the groups with respect to variable *X*. Using a systematic scheme instead, i.e., distributing the units among *T* and *C* in a more balanced way, this may be reduced to *d*_*S*_(*X*).

The crucial question is how such a manipulation affects *d*_*R*_(*Y*), the balance between the groups with respect to another variable. Now there are three types of dependence structures:

A benign dependence structure may be characterized by *d*_*S*_(*Y*) < *d*_*R*_(*Y*). In other words, the effort of balancing *X* pays off, since the increased comparability in this variable carries over to *Y*. For example, given today’s occupational structures with women earning considerably less than men, balancing for gender should also even out differences in income.

If balancing in *X* has no effect on *Y*, *d*_*S*_(*Y*) ≈ *d*_*R*_(*Y*), no harm is done. For example, balancing for gender should not affect the distribution of blood type in the observed groups, since blood type is independent of gender.

Only in the pathological case when increasing the balance in *X* has the opposite effect on *Y*, one may face troubles. As an example, let there be four pairs (*x*_1_, *y*_1_) = (1, 4); (*x*_2_, *y*_2_) = (2, 2); (*x*_3_, *y*_3_) = (3, 1); and (*x*_4_, *y*_4_) = (4, 3). Putting units 1 and 4 in one group, and units 2 and 3 in another, yields a perfect balance in the first variable, but the worst imbalance possible in the second.

However, suppose *d*(⋅) < *c* where the constant (threshold) *c* defines comparability. Then, in the randomized case, the groups are comparable if both *d*_*R*_(*X*) and *d*_*R*_(*Y*) are smaller than *c*. By construction, *d*_*S*_(*X*) ≤ *d*_*R*_(*X*) < *c*, i.e., the systematically composed groups are also comparable with respect to *X*. Given a malign dependence structure, *d*_*S*_(*Y*) increases and may exceed *d*_*R*_(*Y*). Yet *d*_*S*_(*Y*) < *c* may still hold, since, in this case, the “safety margin” *c* − *d*_*R*_(*Y*) may prevent the systematically constructed groups from becoming non-comparable with respect to property *Y*. In large samples, *c* − *d*_*R*_(⋅) is considerable for both variables. Therefore, in most cases, consciously constructed samples will (still) be comparable. Moreover, the whole argument easily extends to more than two factors.

In a nutshell, endeavouring to balance relevant variables pays off. A conscious balancing schema equates known factors better than chance and may have some positive effect on related, but unknown, variables. If the balancing schema has no effect on an unknown factor, the latter is treated as if randomization were interfering—i.e. in a completely nonsystematic, ‘neutral’ way. Only if there is a malign dependence structure, when systematically balancing some variable yields (considerable) “collateral damage”, might randomization be preferable.

This is where sample size comes in. In realistic situations with many unknown nuisance factors, randomization only works if *n* is (really) large. Yet if *n* is large, so are the “safety margins” in the variables, and even an unfortunate dependence structure won’t do any harm. If *n* is smaller, the above models show that systematic efforts, rather than randomization, may yield comparability. Given a small number of units, both approaches only have a chance of succeeding if there are hardly any unknown nuisance factors, or if there is a benign dependence structure, i.e., if a balance in some variable (no matter how achieved) has a positive effect on others. In particular, if the number of relevant nuisance factors and interactions is small, it pays to isolate and control for a handful of obviously influential variables, which is a crucial ingredient of experimentation in the classical natural sciences. Our overall conclusion may thus be summarized in the following table (Table 4):

**Table 4:** Kinds of dependence structures.

Dependence	X (observed)	Y (unobserved)	Preferable procedure
Benign	*d*_*S*_(*X*) < *d*_*R*_(*X*)	*d*_*S*_(*Y*) < *d*_*R*_(*Y*)	Systematic allocation
Neutral	*d*_*S*_(*X*) < *d*_*R*_(*X*)	*d*_*S*_(*Y*) ≈ *d*_*R*_(*Y*)	Systematic allocation
Malign	*d*_*S*_(*X*) < *d*_*R*_(*X*)	*d*_*S*_(*Y*) > *d*_*R*_(*Y*)	Rather systematic than random allocation

## 5 More principled questions

Since randomization has been a core point of dispute between the major philosophical schools of statistics, it seems necessary and appropriate to address these issues here.

### 5.1 The Frequentist position

Possibly the most important, some would say outstanding argument in favour of randomization is the view that the major “function of randomization is to generate the sample space and hence provide the basis for estimates of error and tests of significance” [36]. It has been proposed and defended by prominent statisticians, once dominated the field of statistics, and still has a stronghold in certain quarters, in particular medical statistics, where *randomized* controlled trials have been the gold standard.

In a statistical experiment one controls the random mechanism, thus the experimenter knows the sample space and the distribution in question. This constructed and therefore “valid” framework keeps nuisance variables at bay, and sound reasoning within the paradigm leads to correct results. Someone following this “Frequentist” train of thought could therefore state—and several referees of this contribution have indeed done so—that the above models underline the rather well-known fact that randomization can have difficulties in constructing similar groups (achieving exchangeablility/comparability, balancing covariates), in particular if *n* is small. However, this goal is quite subordinate to the major goal of establishing a known distribution on which quantitative statistical conclusions can be based. More precisely,

randomization in design … is supposed to provide the grounds for *replacing* uncertainty about the possible effects of nuisance factors with a probability statement about error ([37], p. 214, my emphasis).

In other words, because of randomization, all effects of a large (potentially infinite) number of nuisance factors can be captured by a single probability statement. How is this remarkable goal achieved?

Any analytical procedure, e.g., a statistical test, is an algorithm, transferring some numerical input into a certain output which, in the simplest case, is just a number. Given the same data, it yields exactly the same result. The procedure does not go any further: In general, there are no semantics or convincing story associated with a bare numerical result that could increase the latter’s impact. In other words, a strong interpretation needs to be based on the framework in which the calculations are embedded.

Now, since randomization treats all variables (known and unknown) alike, the analytical procedure is able to “catch” them all and their effects show up in the output. For example, a confidence interval, so the story goes, gives a quantitative estimate of all of the variables’ impact. One can thus numerically assess how strong this influence is, and one has, in a sense, achieved explicit quantitative control. In particular, if the total influence of all nuisance factors (including random fluctuations due to randomization) is numerically small, one may conclude with some confidence that a substantial difference between *T* and *C* should be due to the experimental intervention.

Following this line of argument, owing to randomization, a statistical experiment gives a valid result in the sense that it allows for far-reaching, in particular causal, conclusions. Thus, from a Frequentist point of view, one should distinguish between two very different kinds of input: (randomized) experimental data on the one hand and (non-randomized) non-experimental data on the other. Moreover, since randomization seems to be crucial—at least sufficient—for a causal conclusion, some are convinced that it is also necessary. For example, the frequently heard remark that “only randomization can break a causal link” ([38], p. 200) echoes the equally famous statement that there is “no causation without manipulation” [39].

This train of thought is supplemented by the observation that random assignment is easy to implement, and that hardly any (questionable) assumptions are needed in order to get a strong conclusion. For example, Pawitan [40] says:

A new eye drug was tested against an old one on 10 subjects. The drugs were randomly assigned to both eyes of each person. In all cases the new drug performed better than the old drug. The P-value from the observed data is 2^−10^ = 0.001, showing that what we observe is not likely due to chance alone, or that it is very likely the new drug is better than the old one … Such simplicity is difficult to beat. Given that a physical randomization was actually used, very little extra assumption is needed to produce a valid conclusion.

Finally, one finds a rather broad range of verbal arguments why randomization should be employed, e.g. “valid” conclusions, either based on the randomization distribution [41] or some normal-theory approximation [42], removal of investigator bias [43], face validity, fairness, and simple analysis [44], justification of inductive steps, in particular generalizations from the observed results to all possible results [45].

### 5.2 Bayesian opposition

Traditionally, criticism of the Frequentist line of argument in general, and randomization in particular, has come from the Bayesian school of statistics. While Frequentist statistics is much concerned with the way data is collected, focusing on the design of experiments, the corresponding sample space and sampling distribution, Bayesian statistics is rather concerned with the data actually obtained. Its focus is on learning from the(se) data—in particular with the help of Bayes’ theorem—and the parameter space.

In a sense, both viewpoints are perfectly natural and do not contradict one other. However, the example of randomization shows that this cannot be the final word: For the pre-data view, randomization is essential; it constitutes the difference between a real statistical experiment and any kind of quasi-experiment. For the post-data view, however, randomization does not add much to the information at hand, and is ancillary or just a nuisance.

The crucial and rather fundamental issue therefore becomes how far-reaching the conclusions of each of these styles of inference are. To cut a long story short, despite a “valid” framework and mathematically sound conclusions a Frequentist train of thought may easily miss its target or might even go astray. (The long story, containing a detailed philosophical discussion, is told in [46].) After decades of Frequentist—Bayesian comparisons, it has become obvious that in many important situations the numerical results of Frequentist and Bayesian arguments (almost) coincide. However, the two approaches are conceptually completely different, and it has also become apparent that simple calculations within the sampling framework lead to reasonable answers to post-data questions only because of “lucky” coincidences (e.g., the existence of sufficient statistics for the normal distribution). Of course, in general, such symmetries do not exist, and pre-data results cannot be transferred to post-data situations. In particular, purely Frequentist arguments fail if the sampling distribution does not belong to the “exponential family”, if there are several nuisance parameters, if there is important prior information, or if the number of parameters is much larger than the number of observations (*p* ≫ *n*).

### 5.3 A formal as well as an informal framework

The idea that the influence of many nuisance factors—even unknown ones—may be caught by a simple experimental device and some probability theory is a bold claim. Therefore it should come as no surprise that some Frequentist statisticians, many scientists and most Bayesians have questioned it. For example, towards the end of his article, Basu [26] writes quite categorically: “The randomization exercise cannot generate any information on its own. The outcome of the exercise is an ancillary statistic. Fisher advised us to hold the ancillary statistic fixed, did he not?” Basing our inferences on the distribution that randomization creates seems to be the exact reverse.

Even by the 1970s, members of the classical school noted that, upon using randomization and the distribution it entails, we are dealing with “the simplest hypothesis, that our treatment … has absolutely no effect in any instance”, and that “under this *very tight* hypothesis this calculation is obviously logically sound” ([47], my emphasis). Contemporary criticism can be found in Heckman [19] who complains that “a large statistical community” idealizes randomization, “implicitly appeal[s] to a variety of conventions rather than presenting rigorous models”, and that “crucial assumptions about sources of randomness are kept implicit.”

As for the sources of randomness, one should at least distinguish between natural variation and artificially introduced variability. A straightforward question then surely is, how inferences based on the “man-made” portion bear on the “natural” part. To this end, [26], pp. 579–581, compares a scientist, following the logic we described in Section 1, and a statistician who counts on randomization. It turns out that they are talking at cross-purposes. While the foremost goal of the scientist is to make the groups comparable, the statistician focuses on the randomization distribution. Moreover, the scientist asks repeatedly to include important information, but with his inquiry falling on deaf ears, he disputes this statistician’s analysis altogether.

Heckman’s criticism deploring a lack of explicit models and assumptions has been repeated by many (e.g. [31], [7]). In the natural sciences, mathematical arguments have always been more important than verbal reasoning. Typically, the thrust of an argument consists of formulae and their implications, with words of explanation surrounding the formal nucleus. Other fields like economics have followed suit and have learnt—often the hard way—that seemingly very convincing heuristic arguments can be wrong or misleading. Causality is no exception to that rule. In the last twenty years or so, causal graphs and causal calculus have formalized this field. And, as was to be expected, increased rigor straightforwardly demonstrated that certain “reasonable” beliefs and rather “obvious” time-honored conventions do not work as expected (see [7], in particular Chapter 6 and p. 341).

Our analysis above fits in nicely: The standard phrase “if *n* is not too small” is a verbal statement, implicitly appealing to the central limit theorem. Owing to the latter theorem, groups created by random assignment tend to become similar. The informal assurance, affirming that this happens fast, ranks among the most prominent conventions of traditional statistics. However, explicit numerical models underline that our intuition needs to be corrected. Rather straightforward calculations suffice to show that fluctuations cannot be dismissed easily, even if *n* is large.

Worse still, the crucial part of the Frequentist’s main argument in favour of randomization is informal in a rather principled way: In an experimental, as well as in a similar non-experimental situation, the core formal machinery, i.e. the data at hand, the explicit analytical procedure (e.g. a statistical test), and the final numerical result may be identical. In other words, it is just the *narrative* prior to the data that makes such a tremendous difference in the end. Since heuristic arguments have a certain power of persuasion which is certainly weaker than a crisp formal derivation or a strict mathematical proof, it seems to be no coincidence that opinion on this matter has remained divided. Followers of Fisher believed in his intuition and trusted randomization; critics did not. And since, sociologically speaking, the Frequentist school dominated the field for decades, so did randomization.

It is also no coincidence, but rather sheer necessity, that a narrow formal line of argument needs to be supplemented with much intuition and heuristics. So, on the one hand, an orthodox author may claim that “randomization, instrumental variables, and so forth have *clear* statistical definitions”; yet, on the other hand, he has to concede at once that “there is a long tradition of *informal*—but systematic and successful—causal inference in the medical sciences” ([7], p. 387, my emphasis). Without doubt, such a mixture is difficult to understand, to use and to criticize, and could be one of the main reasons for the reputation of statistics as an opaque subject. The narrow formal framework also partly explains why there is such a wide variety of verbal arguments in favour of randomization (see the end of Section 5.1).

### 5.4 Pragmatical eclecticism

From a Frequentist point of view, randomization is crucial since it “provides a known distribution for the assignment variables; statistical inferences are based on this distribution” [48]. Thus, the “known-distribution argument” is perhaps the single most important argument in favour of randomization.

How is it applied? If the result of a random allocation is extreme (e.g. all women are assigned to T, and all men to C), everybody—Fisher included—seems to be prepared to dismiss this realization: “It should in fairness be mentioned that, when randomization leads to a bad-looking experiment or sample, Fisher said that the experimenter should, with discretion and judgement, put the sample aside and draw another” [24].

The latter concession isn’t just a minor inconvenience, but runs contrary to the very principle of the Frequentist viewpoint: First, an informal correction is wide open to subjective judgement. (Already) “bad-looking” to person A may be (still) “fine-looking” to person B. Second, what’s the randomization distribution actually being used when dismissing some samples? A vague selection procedure will inevitably lead to a badly defined distribution. Third, why reject certain samples at all? If the crucial feature of randomization is to provide a “valid” distribution (on which all further inference is based), one should not give away this advantage unhesitatingly. At the very least, it is inconsistent to praise the argument of the known framework in theoretical work, and to turn a blind eye to it in practice.

As a matter of fact, in applications, the exact permutation distribution created by some particular randomization process plays a rather subordinate role. Much more frequently, randomization is used as a rationale for common statistical procedures. Here is one of them: Randomization guarantees independence and if many small uncorrelated (and also often unknown) factors contribute to the distribution of some observable variable *X*, this distribution should be normal—at least approximately, if *n* is not too small. Therefore, in a statistical experiment, it seems to be justified to compare ${\bar{X}}_{T}$ and ${\bar{X}}_{C}$, using these means and the sample variance as estimators of their corresponding population parameters. Thus we have given an informal derivation of the t-test. Both the test and the numerical results it yields are supported by randomization. However, it may be noted that Student’s famous test was introduced much earlier [49] and worked quite well without randomization’s assistance.

How should experimental data be analyzed? If the known distribution were of paramount importance, there should be a unanimous vote, at least by Frequentist statisticians. However, only a minority, perfectly in line with the received position, advise leaving the data as it is. Freedman [50] argues thus (for similar comments see [48], [7], p. 340, and [38], pp. 250–253.):

Regression adjustments are often made to experimental data. Since randomization does not justify the models, almost anything can happen … The simulations, like the analytic results, indicate a wide range of possible behavior. For instance, adjustment may help or hurt.

Yet a majority have a different opinion (e.g. [2, 3, 45]). Tu et al. [51] explain that the first reason why they opt for an “adjustment of treatment effect for covariates in clinical trials” is to “improve the credibility of the trial results by demonstrating that any observed treatment effect is not accounted for by an imbalance in patient characteristics.”

### 5.5 Once again: randomization vs. comparability

Apart from the rather explicit rhetoric of a “valid framework”, there is also always the implicit logic of the experiment. Thus, although the received theory emphasizes that “actual balance has nothing to do with validity of statistical inference; it is an issue of efficiency only” [41]; comparability turns out to be crucial:

Many, if not most, of those supporting randomization rush to mention that it promotes similar groups. Nowadays, only a small minority bases its inferences on the known permutation distribution created by the process of randomization; but an overwhelming majority checks for comparability. Reviewers of experimental studies routinely request that authors provide randomization checks, that is, statistical tests designed to substantiate the equivalence of *T* and *C*. At least, in almost every article a list of covariates—with their groupwise means and standard errors—can be found.

A narrow, restricted framework is only able to support weak conclusions; there is no such thing as a “free lunch.” Therefore, upon reaching a strong conclusion, there must be implicit, hidden assumptions (cf. [19], pp. 139, 155). In particular, a second look at the “little-assumption” argument reveals that it is the hidden assumption of comparability that carries much of the burden of evidence: It is no coincidence that in Pawitan’s example an eye drug was tested. Suppose one had tested a liver drug instead. The same numerical result would be almost as convincing if such a drug had been applied to twins. However, if the liver drug had been administered to a heterogenous set of persons or if it had been given to a different biological species (mice instead of men, say), exactly the same formal result would not be convincing at all; since, rather obviously, a certain discrepancy a priori may cause a remarkable difference a posteriori.

Savage’s example is quite similar. No matter how one splits a small heterogenous group into two, the latter groups will always be systematically different. Randomization does not help: If you assign randomly and detect a large effect in the end, still, your experimental intervention *or* the initial difference between *T* and *C* may have caused it. All “valid” inferential statistics is, in a sense, an illusion, since it cannot exclude the straightforward second explanation. Instead, it’s the initial exchangeability of the groups that turns out to be decisive; similarity of *T* and *C* rules out the second explanation and leaves the experimental intervention as the only cause.

In conclusion, comparability, much more than randomization, keeps alternative explanations at bay. Since it is our endeavour to achieve similar groups, minimization is not just some supplementary technique to improve efficiency. Rather, it is a straightforward and elaborate device to enhance comparability, i.e., to consciously construct similar groups. (The influence of unknown factors is discussed in Section 4.) Though, at times, we fail, e.g. “it does not seem possible to base a meaningful experiment on a small heterogenous group” [23], there can hardly be any doubt that “the purpose of randomization is to achieve homogeneity in the sample units. [Thus] it should be spelled out that stability and homogeneity are the foundation of the statistical solution, *not* the other way around” [52], p. 70 (my emphasis).

In a nutshell, nobody, not even Fisher, follows “pure Frequentist logic”, in particular the distribution that randomization generates. In a strict sense, there is no logic at all, rather a certain kind of mathematical reasoning plus—since the formal framework is restricted to sampling—a fairly large set of conventions; rigid “pure” arguments being readily complemented by applied “flexibility”, consisting of time-honored informal reasoning and shibboleth, but also outright concessions. Bayesians noticed long ago [28, 31] that “Frequentist theory is shot full of contradictions” [53], and during the last few decades, efforts to overcome the received framework have gained momentum.

## 6 A broader perspective

In the 20th century, R.A. Fisher (1890–1962) was the most influential statistician. However, while his early work on mathematical statistics is highly respected in all quarters, hardly anybody relies on his later ideas, in particular fiducial inference [24]. Randomization lies in-between, and, quite fittingly, public opinion on this formal technique has remained divided.

### 6.1 Replicate!

Since the most important application of random assignment can be found in clinical trials, it is straightforward to ask how strong the evidence produced by a randomized controlled trial is. Vis-à-vis the rather anecdotal and qualitative research that preceded today’s RCTs, the latter surely constituted real progress. Strict design and standardized analysis has raised the bar and has fostered consensus among researchers. However, many classical experiments in the natural sciences are deterministic and have an even better reputation. If in doubt, physicists do not randomize, but replicate. Fisher [54], p. 58, gave similar advice.

Alas, a large number of important biomedical findings (RCTs included) failed this examination and turned out to be non-replicable [55–57], so that the National Institute of Health was forced to launch the “Replication of Key Clinical Trials Initiative” [58]. The same with experimental psychology which has relied on (small) randomized trials for decades. Now, it is lamenting a “replicability crisis” that has proved to be so severe that an unprecedented “reproducibility project” needed to be launched [59, 60]. (Similar initiatives are collected in http://validation.scienceexchange.com. One may also consult www.nature.com/nature/focus/reproducibility on this matter.)

The logic of Section 1 offers a straightforward explanation for this unfortunate state of affairs. Given (almost) equal initial conditions, the same boundary conditions thereafter, and a well-defined experimental intervention, an effect once observed must re-occur. That’s how classical experiments work, which reliably and thus repeatedly hit their target. During the experiment, a controlled environment keeps disturbing factors at bay. Thus, if an effect cannot be replicated, the constructional flaw should be sought at the very beginning of the endeavour. At this point, it is conspicuous that today’s studies do not focus explicitly on the crucial idea of comparability. With other issues—possibly rather irrelevant or even misleading—being at least as important, initial imbalances are the rule and not the exception. At the very least, with randomization, the starting point of researcher 2, trying to repeat the result of researcher 1, will always differ from the latter’s point of origin. Therefore, if an effect cannot be replicated, this may well be due to the additional variability introduced by the “R” in RCT, yielding unequal initial conditions, and “drowning” the interesting phenomenon in a sea of random fluctuation. In a similar but more positive vein, Abel and Koch [16] underline that experimental control (the “C” in RCT) is crucial:

Apart from the control of imbalance in prognostic factors, randomized studies have several qualities that … follow from the fact that [they] belong to a larger class of high-quality studies, namely, prospective parallel comparisons with a written protocol specifying important aspects of patient enrollment, treatment, observation, analysis, and other procedures.

### 6.2 Learning to cope with variability

Chance has a Janus face. The idea that many (the more the better), small and rather uncorrelated random influences sum up to a “mild” distribution originated in the 19th century, culminating in the famous central limit theorem on which much of classical statistics is built. However, this was not the end of the story. Studying complex systems, physicists soon encountered “wild” distributions, in particular power laws ([61], p. 104). It is well within this newer set of ideas that a single random event may have a major impact that cannot be neglected (e.g. the energy released by a particularly strong earthquake or the area devastated by a single large flood). The fact that the process of randomization can produce a major fluctuation, i.e., a pronounced imbalance in a covariate (and thereby between *T* and *C*), exerting a tremendous influence on the final result of an RCT is in line with this more recent portrait of chance.

Randomization has tremendous prestige in orthodox statistics, downgrading all designs without a random element to quasi-experiments. One even distinguishes between truly random allocation and “haphazard assignment, that is, a procedure that is not formally random but has no obvious bias” ([3], p. 302). Honoring thus the classical philosophical distinction between “deterministic” and “random‘”, one readily neglects the fact that modern dynamical systems theory sees a continuum of increasing complexity between perfect (deterministic) order and “randomness [which] can be thought of as an extreme form of chaos” [62].

With the core technique (or rather dogma) of randomization, Fisher’s conception of experiments could even develop into a “cult of the single study” ([63], p. 262), and catch-phrases highlighting randomization’s outstanding role became increasingly popular [38, 39]. However, this determined point of view has also blocked progress, and innovative solutions have been developed elsewhere: Econometrician J.J. Heckman, earning a Nobel prize for his contributions in 2000, explains that Holland’s claim that there can be no causal effect of gender on earnings (because we cannot randomly assign gender) “conflates the act of definition of the causal effect … with empirical difficulties in estimating it” [19]. Moreover, he complains that “this type of reasoning is prevalent in statistics.” Epidemiology, not following Fisher [64] but the Advisory Committee to the Surgeon General of the Public Health Service [65], has made its way to causal graphs. The latter formalization, mainly developed by computer scientist J. Pearl, has given crucial concepts a sound basis, but it also tells a “tale of statistical agony” [7].

In a more positive vein, computer scientist J. Rissannen [66] showed how Fisher’s finest ideas may be reformulated and extended within a modern, fine-tuned mathematical framework. In his work one finds a logically sound and general unifying theory of hypothesis testing, estimation and modeling; yet there is no link to randomization. Instead, in this contemporary theory the basic concept is Kolmogorov complexity, allowing to express the idea that a regular sequence **r** (e.g. “1,0” repeated 13 times) is less complex than a sequence like **s** = (1, 0, 0, 1, 0, 0, 1, 1, 0, 1, 1, 0, 0, 0, 1, 1, 1, 0, 1, 1, 0, 1, 0, 0, 0, 0) in a mathematically strict way. (The sequence **s** can be found in [67], p. 48; a book including a chapter on “nonprobabilistic statistics.”) It also turns out that stochastic processes typically produce complex sequences. However, contrary to the fundamental distinction (deterministic vs. random) mentioned above, given a certain sequence like **s**, it is not possible to tell whether the process that generated **s** was systematic or not. **s** could be the output of a “(pseudo-)random number generator”, i.e., a deterministic algorithm designed to produce chaotic output, or of a “truly random” mechanism. (Whatever that is. For example, strictly speaking, coin tossing—being a part of classical physics—is not “truly” random.)

Interpreting **s** as a particular assignment of units to groups (1 → *T*, and 0 → *C*, say), the above fundamental distinction between “‘haphazard” and “random” assignment processes seems to be exaggerated, some might even question whether it is relevant at all. However, it is difficult to deny that the (non-)regularity of the concrete mapping matters. Just compare **r** to **s**: Since **r** invites a straightforward alternative explanation, most people would prefer **s**. In today’s terminology, Fisher could have been looking for maximally complex sequences, i.e., allocations without any regularity. In his time, a simple stochastic process typically yielding an “irregular” sequence was a straightforward and convenient solution to this problem.

### 6.3 Conclusion: Good experimental practice

In sum, Fisher’s idea of randomization is still alive. However, on the whole it looks more like a backward-looking tool than like the indispensable key to the future of statistics. Though there are many claims in its favour, they can all be seriously questioned:

1. Random assignment makes groups comparable.
  Yes, but only if n is large. Otherwise, randomization rather provokes imbalances (Section 3).
2. A random process treats known and unknown factors alike, and thus controls unknown nuisance factors.
  Conversely, imbalances in observed variables hint at imbalances in unobserved variables. Moreover, a more detailed study of dependence structures reveals that consciously working in favour of similar groups typically pays off (Section 4).
3. The act of randomization generates the sample space and hence provides the basis for valid conclusions.
  The formal Frequentist framework thus defined is narrow, yielding weak conclusions that have to be supplemented with a lot of informal reasoning (Section 5).
4. Random allocation allows for causal inference since it keeps nuisance factors at bay.
  No; comparability and strict experimental control are crucial for internally valid, replicable studies (Section 6).

In a nutshell, contrary to what Fisher [1] thought, randomization does *not* “relieve the experimenter from the anxiety of considering and estimating the magnitude of the innumerable causes by which the data may be disturbed.” Quantitative arguments and formalized theories demonstrate that it is no philosopher’s stone, almost effortlessly lifting experimental procedures in the medical and social sciences to the level of classical experiments in the natural sciences. Rather, random assignment may lull researchers into a false sense of security.

It is true that chance, in the guise of randomization, by and large supports comparability. However, since the former is blind with respect to the concrete factors and relevant interactions that may be present, it needs a large number of experimental units to do so. The intuition behind this result is easy to grasp: Without knowledge of the subject matter, randomization has to protect against every conceivable nuisance factor. Such unsystematic protection is provided by number and builds up slowly. Thus, a huge number of randomly allocated subjects is needed to shield against a moderate number of potential confounders. And, of course, no finite procedure such as the flip of a coin is able to control for an infinite number of nuisance variables.

Therefore, it seems much more advisable to use background knowledge in order to minimize the difference between groups with respect to known factors or specific threats to experimental validity. As of today, minimization seems to operationalize this idea best. At the end of such a conscious construction process, randomization finds its proper place. Only if no reliable context information exists is unrestricted randomization the method of choice. It must be clear, however, that it is a weak guard against confounding, yet the only one available in such inconvenient situations.

Summing up, the above analysis strongly recommends traditional experimentation, thoroughly selecting, balancing and controlling factors and subjects with respect to known relevant variables, thereby using broader context information—i.e., substantial scientific knowledge. I agree with Penston [68], pp. 76–77, who says:

… it is the existence of sound background theory which is crucial for the success of science. It is the framework against which observations are made, it allows strict definition of the items involved, it is the source of information about possible relevant variables and allows for the identification of homogeneous reference classes that ensure regularity and, hence, reliable causal inference.

Cumulative science is the result of a successful series of such experiments, each of them focusing on the crucial ingredients, like precise research questions, convincing operationalizations, explicit control, quantitative measures of effect, and—last but not least—comparability.
